# Efficacy and safety of low-dose triple and quadruple combination pills for initial hypertension management: a systematic review and meta-analysis of RCTs

**DOI:** 10.1097/MS9.0000000000005722

**Published:** 2026-10-13

**Authors:** Asad Ali Ahmed Cheema, Syed Moiz Abbas, Laiba Nadeem, Hafsa Azam, Ahmed Muneeb Rashid, Khadija Alam, Kumail Mustafa Ali, Abdur Rafay Bilal, Muzamil Hamid Hussain, Chaudhry Saad Sohail, Syed Sarmad Javaid

**Affiliations:** a Department of Medicine, International School of Medicine, International University of Kyrgyzstan, Bishkek, Kyrgyzstan; b Department of Medicine, Jinnah Sindh Medical University, Karachi, Pakistan; c Department of Medicine, Karachi Medical and Dental College, Karachi, Pakistan; d Department of Medicine, Dow University of Health Sciences, Karachi, Pakistan; e Department of Medicine, Liaquat National Hospital and Medical College, Karachi, Pakistan; f Department of Medicine, Ziauddin Medical College, Karachi, Pakistan; g Department of Internal Medicine, University of Mississippi Medical Center, Jackson, United States; h Department of Medicine, University of Mississippi Medical Center, Jackson, United States

**Keywords:** blood pressure control, hypertension, low-dose combination therapy, polypill, triple or quadruple therapy

## Abstract

**Background:**

Hypertension is a leading modifiable risk factor for cardiovascular disease, yet global blood pressure (BP) control remains suboptimal. A low-dose combination (LDC) polypill may simplify therapy and enhance BP reduction. We synthesized randomized evidence on the efficacy and safety of initial LDC triple and quadruple antihypertensive pills.

**Methods:**

PubMed, ClinicalTrials.gov, WHO ICTRP, and the Cochrane Library were searched from inception to 20 May 2026. Randomized controlled trials (RCTs) comparing LDC triple- or quadruple-pill regimens with active comparators (monotherapy or usual care) or placebo were included. Random-effects meta-analyses estimated odds ratios (ORs) and mean differences (MDs) with 95% confidence intervals (CIs).

**Results:**

Fourteen RCTs (*n* = 4082) were included. Versus active comparators, LDC increased achievement of target BP at 4–12 weeks (OR: 1.90; 95% CI: 1.40–2.57) and 6–12 months (OR: 1.76; 95% CI: 1.39–2.24). LDC reduced systolic BP (−3.33 mmHg; 95% CI: −6.59 to −0.06) and diastolic BP (MD: −2.71 mmHg; 95% CI: −4.57 to −0.86) versus active comparators, and lowered systolic BP versus placebo (−10.93 mmHg; 95% CI: −18.06 to −3.80). Overall adverse events were similar between groups, but dizziness/syncope/presyncope was slightly more frequent with LDC (OR: 1.41; 95% CI: 1.02–1.95). Headache was less common versus placebo (OR: 0.27; 95% CI: 0.08–0.95). Lipids and creatinine were unchanged; sodium (MD: −0.72 mmol/L) and potassium (MD: −0.19 mmol/L) decreased modestly.

**Conclusion:**

Initial LDC triple and quadruple pills improve BP control and are generally well tolerated, although a modest increase in dizziness was observed.

## Introduction

Hypertension is the leading modifiable risk factor for cardiovascular disease and premature death worldwide. Despite the widespread availability of effective treatment regimes, only about 21% of individuals with hypertension have their condition adequately controlled according to the World Health Organization (WHO)^[^1^]^. Uncontrolled blood pressure (BP) significantly increases the risk of stroke, myocardial infarction, heart failure, and chronic kidney disease (CKD)^[^2^]^. The global burden of hypertension is expected to rise further due to aging populations, urbanization, sedentary lifestyles, and dietary changes, particularly in developing nations^[^3^]^.

HIGHLIGHTS

- Initial low-dose combination therapy (LDC) significantly increased the proportion of patients achieving target blood pressure compared with monotherapy or placebo.
- LDC triple or quadruple regimens produced greater reductions in systolic and diastolic blood pressure, with benefits sustained over short- and mid-term follow-up.
- The safety profile of LDC therapy was comparable to active comparators and placebo, with no meaningful increase in adverse events, serious events, or treatment discontinuation.
- Laboratory parameters remained largely stable, with only minor, clinically non-significant electrolyte changes.
- Overall findings support initial LDC therapy as an effective and well-tolerated strategy for improving blood pressure control, while underscoring the need for longer-term studies in diverse and high-risk populations.

Initiating effective BP control early in the course of hypertension is critical as it significantly reduces the long-term risk of developing serious cardiovascular and cerebrovascular complications. Evidence shows that earlier and more intensive BP control leads to better outcomes, especially in high-risk individuals^[^4,5^]^. Studies have demonstrated that most patients require two or more antihypertensive agents to achieve their target BP, suggesting that monotherapy may be insufficient for many individuals from onset^[^6^]^.

However, poor medication adherence remains one of the most significant barriers to effective BP management. Studies show that up to 50% of patients discontinue antihypertensive therapy within 1 year of initiation, often due to complex multi-pill regimens and treatment fatigue^[^7^]^. Furthermore, the lack of timely escalation or adjustment of treatment when optimal targets are not achieved further hinders effective BP control in real-world clinical practice^[^8^]^.

Previously, it was proposed that administering antihypertensive agents at low doses could retain most of their BP–lowering benefits while reducing adverse effects, and that combining drugs from different pharmacological classes results in additive therapeutic effects^[^9,10^]^. Recently, low-dose combination (LDC) therapy, particularly in the form of a polypill, is emerging as a promising solution. It involves using mostly three or more drug classes of antihypertensive agents at reduced doses in a single pill. The polypill simplifies treatment regimens, enhances adherence, and targets multiple physiological mechanisms simultaneously, thereby achieving greater reductions in BP than monotherapy, even at low doses^[^10^]^. LDC therapy may also help overcome therapeutic inertia and facilitate adherence – two key drivers of poor hypertension control worldwide^[^11–13^]^. Further, evidence from recent trials have demonstrated that LDC therapy not only reduces both systolic and diastolic BP significantly, but also improves other cardiometabolic parameters including mean heart rate, low-density lipoprotein (LDL) cholesterol, with comparable or even fewer side effects compared to placebo or multiple separate pills^[^14,15^]^.

A previous meta-analysis provided valuable insights into the efficacy and safety of LDC therapies for the initial management of hypertension^[^16^]^. However, the current evidence remains limited and somewhat inconclusive, largely due to the small number and modest sample sizes of available trials. Most included studies have primarily assessed short-term BP outcomes, with less attention to safety profiles, laboratory parameters, and long-term clinical effects. Importantly, several new randomized controlled trials (RCTs) have been published since earlier reviews, highlighting the need for an updated synthesis to incorporate these findings and better inform clinical practice. This updated analysis not only revisits the previously synthesized outcomes but also expands the scope by incorporating data on safety profiles and laboratory parameters. Henceforth, we conducted this updated systematic review and meta-analysis to evaluate the efficacy and safety of LDC therapy along with their corresponding laboratory parameters compared to active comparator (initial monotherapy or usual care) or placebo as the initial treatment for hypertension.

## Methods and materials

This systematic review and meta-analysis followed the Preferred Reporting Items for Systematic Reviews and Meta-Analyses (PRISMA) guidelines^[^17^]^ and was registered on PROSPERO [CRD420261323534]. Ethical review board approval was not required, as the data used were publicly available.

### Literature search

A systematic search was performed up to 20 May 2026 on major electronic databases including PubMed, Cochrane Library, ClinicalTrials.gov and WHO ICTRP for studies evaluating LDC triple or quadruple therapy for initial management of hypertension. MeSH terms like “Hypertension” or “Drug therapy combination” or “Single dose pill” combined with Boolean operators were used to run the literature search. A detailed search string for each database has been provided in Supplemental Digital Content Table S1, available at: https://links.lww.com/MS9/B412. Additionally, reference lists of included articles, such as trials, reviews, and meta-analyses, were manually screened to identify any relevant studies missed in the electronic search. The literature search was exclusively conducted in English, while studies in any other language were excluded. Search results were uploaded to Rayyan, a web-based screening platform, and screened independently by two reviewers^[^18^]^. Titles, abstracts, and full texts were reviewed to exclude duplicates and studies not meeting the eligibility criteria. Disagreements were resolved through discussion with a third investigator. There were no restrictions with regards to country, follow-up period, or year of publication.

### Study selection

Eligible studies met the following inclusion criteria: (1) Study type: RCTs or clinical trials; (2) Population: patients with hypertension, with or without baseline antihypertensive treatment; (3) Intervention: initial LDC triple or quadruple antihypertensive therapy; (4) Comparator: active comparator (initial monotherapy or usual care) or placebo; (5) Outcomes: (a) change in systolic and diastolic BP from baseline; (b) percentage of patients achieving a clinical BP target of <140/90 mm Hg, or a target of <130/80 mm Hg for home BP or for patients with diabetes or CKD, at 4–12 weeks and 6–12 months; (c) adverse, serious adverse, and specific adverse events, including dizziness, syncope, headache, musculoskeletal pain, all types of hypotension, pruritus, peripheral edema, and chest pain; and (d) change in laboratory parameters, including serum levels of triglycerides (mg/dL), creatinine (mg/dL), alanine aminotransferase (ALT) (U/L), aspartate aminotransferase (AST) (U/L), sodium, and potassium (mmol/L). Editorials, conference abstracts, reviews, case reports, case series, and studies with insufficient data or no definitive comparison were excluded.

### Data extraction and quality assessment

Two authors independently performed the process of extracting data from relevant studies. Data extracted included first author, publication year, study location, study setting, number of participants per group, mean age, number of male participants, and treatment regimens for both the study arms. Baseline and follow-up measurements of BP and relevant laboratory parameters were also collected. We also extracted odds ratios (ORs) for the binary outcomes and mean difference (MD) for continuous outcomes along with their 95% confidence intervals (CIs). The quality of included studies was independently assessed by two reviewers using the Revised Cochrane Risk-of-Bias Tool for Randomized Trials (RoB 2)^[^19^]^. This instrument evaluates potential bias across five domains: randomization process, deviations from intended interventions, missing outcome data, measurement of outcomes, and selection of the reported result. Each domain was classified as low risk, some concerns, or high risk. Discrepancies in assessments were resolved by consensus.

### Statistical analysis

For the analysis, LDC therapy (either triple or quadruple combinations) was compared with placebo or an active comparator (initial monotherapy or usual care). We calculated the OR using the random-effects model and the Mantel-Haenszel method, and we estimated the MD with a 95% CI using the inverse variance for continuous data. Forest plots were created to visually assess the results of pooling. The Higgin’s *I*^2^ test assessed the heterogeneity as low (25%), moderate (25–75%), or high (>75%). All statistical analyses were carried out utilizing RevMan software (Review Manager Version 5.3.5, The Nordic Cochrane Centre, Copenhagen). A *P*-value of <0.05 was considered statistically significant. To evaluate the potential influence of each study on the overall outcome, a leave-one-out sensitivity analysis was also performed.

### Grading the evidence

The certainty of evidence was assessed using the Grading of Recommendations Assessment, Development, and Evaluation (GRADE) tool^[^20^]^. Certainty was systematically evaluated on pre-defined criteria and downgraded for risk of bias, inconsistency, indirectness, and imprecision^[^20^]^. Certainty for each outcome was then categorized as high, moderate, low, or very low.

## Results

A total of 10 037 articles were retrieved through the initial search. After removing duplicates and records marked as ineligible or for other reasons, 9228 articles were excluded. After primary screening via title and abstract, 654 studies were excluded based on predefined inclusion and exclusion criteria. After the full-text assessment of the remaining studies, 14 studies evaluating the initial LDC therapy for hypertension were selected. The PRISMA flowchart summarizing the study selection process is given in Figure 1 and the PRISMA checklist has been provided in Supplemental Digital Content Table S2, available at: https://links.lww.com/MS9/B412. For the present updated meta-analyses, data of new studies were synthesized together with the data from the previous reports.

**Figure 1.**
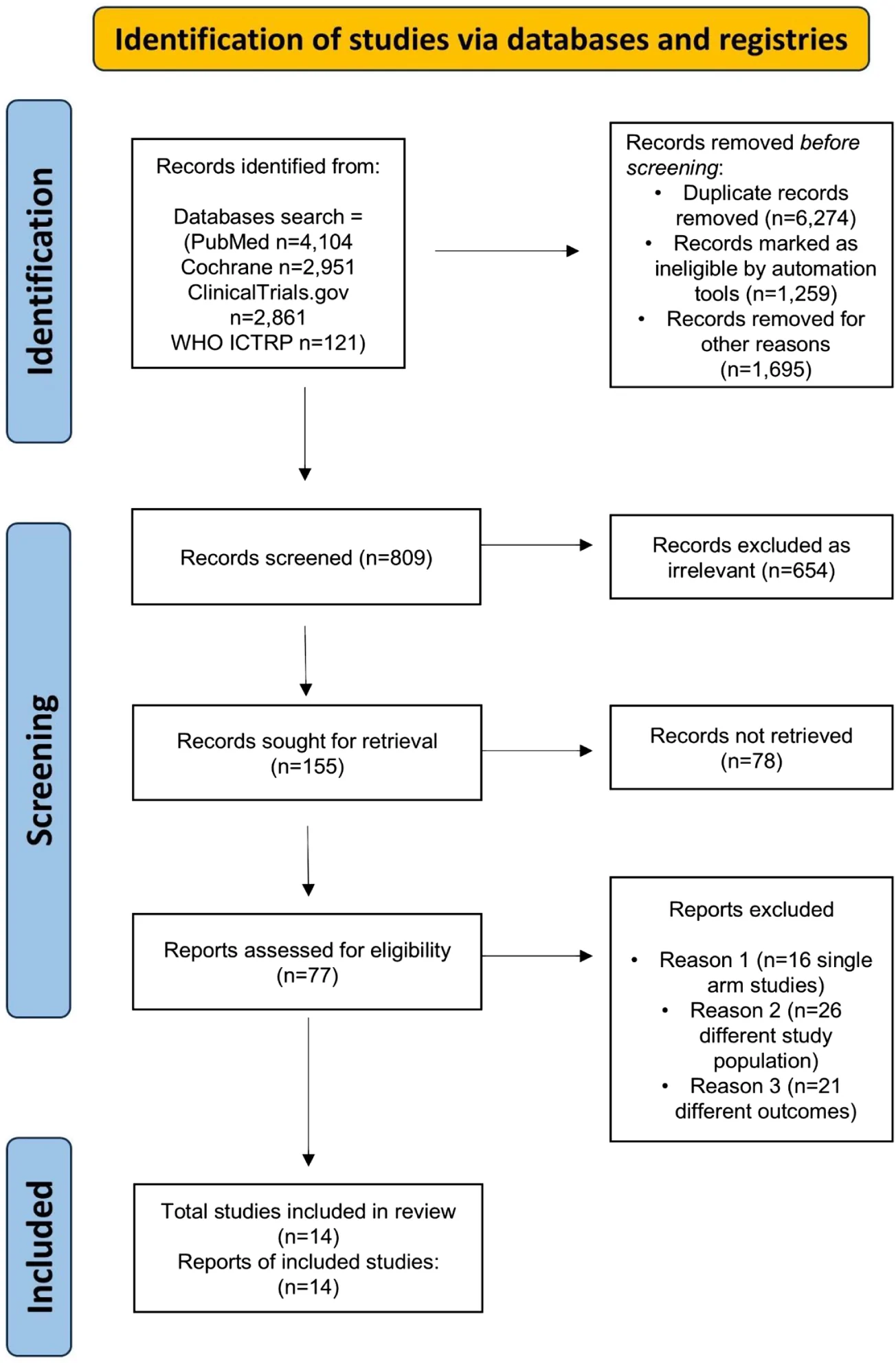
PRISMA flow chart summarizing the study selection process.

### Study and baseline characteristics

Overall, fourteen RCTs with a total of 4082 patients with hypertension were analyzed^[^21–33^]^ and^[^34^]^. The included RCTs demonstrated considerable geographic variation, with studies originating from Nigeria, United States, Australia, China, Sri Lanka, Australia, Korea, Tanzania and Lesotho. Seven RCTs utilized triple combination regimen^[^21,24,26,27,29,30,34^]^ while seven studies used quadruple combination^[^22,23,25,28,32,33,35^]^. Three included studies employed a crossover randomized controlled design^[^24,31,32^]^. In the included trials, triple-therapy regimens predominantly comprised an angiotensin II receptor blocker, a calcium channel blocker, and a diuretic, whereas quadruple-therapy regimens additionally incorporated either a beta-blocker or an angiotensin-converting enzyme inhibitor, with occasional inclusion of a statin. Among these, 1849 patients received LDC triple or quadruple therapy, 1885 received the active comparator (i.e. monotherapy or usual care), 151 received placebo and 197 received both LDC therapy and Placebo. The mean age of the patients was 55.06 years and 49.92% (*n* = 2038) were males. Out of these, 10.09% (*n* = 408) of patients had diabetes and 36.3% (*n* = 1482) were on baseline antihypertensive medication. The detailed baseline characteristics of each study have been summarized in Table 1.

**Table 1:** Baseline characteristics of the included studies.

Study	Study location	Study design	Sample Size	Mean age, years	Male gender (n/N)	Antihypertensives at baseline No. (%)	LDC triple or quadruple therapy	Comparator	Baseline BP, mmHg	Follow-up (months)
Webster 2018^[^21^]^	Sri Lanka	RCT	700	56	297/700	287 (41)	Telmisartan, 20 mg; amlodipine, 2.5 mg; chlorthalidone 12.5 mg	Usual care	154/90	6
Chow 2021^[^22^]^	Australia	RCT	591	59	356/591	273 (46)	Irbesartan, 37.5 mg; amlodipine, 1.25 mg; indapamide, 0.625 mg; bisoprolol, 2.5 mg	Irbesartan, 150 mg	152/88	3
Mahmud 2007^[^23^]^	Ireland	RCT	108	50	70/108	0	Amlodipine, 1.25 mg; atenolol, 12.5 mg; bendroflumethiazide, 0.625 mg; captopril, 12.5 mg	Amlodipine, 5 mg, or atenolol, 50 mg, or bendroflumethiazide, 2.5 mg, or captopril, 100 mg	160/96	1
Hong 2020^[^24^]^	Korea	RCT	238	64	166/238	0	Losartanm, 25 mg; amlodipine, 2.5 mg; chlorthiadone, 6.25 mg	Amlodipine, 5 and 10 mg; losartan, 100 mg; placebo	154/91	2
Chow 2017^[^25^]^	Australia	RCT	21	58	10/21	0	Irbesartan, 37.5 mg; amlodipine, 1.25 mg; hydrochlorothiazide, 6.25 mg; atenolol, 12.5 mg	Placebo	154/90	1
Sung 2022^[^26^]^	Korea	RCT	176	60	64/86	158 (90)	Telmisartan, 20 mg; amlodipine, 2.5 mg; chlorthiadone, 6.25 mg	Amlodipine, 5 and 10 mg; losartan, 100 mg; placebo	151/92	2
Ojji 2024^[^27^]^	Nigeria	RCT	300	52	138/300	NA	combination of telmisartan, amlodipine, and indapamide in triple one-quarter, one-half, and standard doses	standard-care protocol starting with amlodipine (5 mg)	156/97 mm Hg	6
Huffman 2024^[^28^]^	USA	RCT	62	52	34/62	52 (83.9)	Candesartan 2 mg, amlodipine 1.25 mg, indapamide 0.625 mg, and bisoprolol 2.5 mg	candesartan 8 mg	138.1 /84.3	3
Rodgers 2024^[^29^]^	USA	RCT	295	51	44/100	138 (46.77)	GMRx2 ¼ dose (telmisartan 10 mg/amlodipine 1.25 mg/indapamide 0.625 mgGMRx2 ½ dose (telmisartan 20 mg/amlodipine 2.5 mg/indapamide 1.25 mg)	Placebo	138/86	1
Mapesi 2025^[^30^]^	Lesotho and Tanzania.	RCT	758	54	208/758	NA	Three-pill strategy (Amlodipine 2.5 mg OD + Losartan 12.5 mg OD + Hydrochlorothiazide 6.25 mg OD)	Stepped monotherapy strategy (Amlodipine 10 mg OD)	NA	3
Wald 2012^[^31^]^	India	RCT	86	59	64/86	86 (100)	Amlodipine, 2.5 mg; losartan, 25 mg; hydrochlorothiazide, 12.5 mg; simvastatin, 40 mg	Placebo	143/86	3
Zhao 2025^[^32^]^	China	RCT	90	44	67/90	4 (4.4)	Quadruple pill (irbesartan 75 mg, metoprolol 23.75 mg (metoprolol tartrate sustained-release tablet), amlodipine 2.5 mg, indapamide 1.25 mg in total [referred to as “1/2 (A + B + C + D)”]	Standard-dose dual capsule [irbesartan 150 mg and amlodipine 5 mg in total (“A + C” for short]	NA	1
Hu 2021^[^33^]^	China	RCT	60	47	42/60	13 (21.67)	Quadruple pill (reserpine 0.1 mg, dihydralazine 12.5 mg, hydrochlorothiazide 12.5 mg, and triamterene 12.5 mg)	Placebo	141/90	3
Sung 2026 (A)^[^34^]^	South Korea	RCT	352	61	209/352	254 (72.16)	LDC: single-pill combination of amlodipine 1.67 mg losartan potassium 16.67 mg chlorthalidone 4.17 mg	Standard-dose mono therapy – 5 mg amlodipine	NA	2
Sung 2026 (B)	South Korea	RCT	245	62	132/245	217 (88.57)	LDC: single-pill combination of amlodipine 1.67 mg losartan potassium 16.67 mg chlorthalidone 4.17 mg	Standard-dose mono therapy – 50 mg losartan potassium	NA	2

RCT, randomized control trial; LDC, low-dose combination; BP, blood pressure.

### Primary outcomes

#### Proportion of patients achieving target BP

Our pooled analyses demonstrate that LDC triple or quadruple therapy significantly increased the proportion of patients achieving BP targets. At 4–12 weeks, nine studies (3138 patients) reported higher odds versus active comparators (OR: 1.90; 95% CI: 1.40–2.57; *P* < 0.0001; *I*^2^ = 73%, Fig. 2A), while five studies (624 patients) showed an even greater effect versus placebo (OR: 5.94; 95% CI: 3.03–11.65; *P* < 0.00001; *I*^2^ = 43%, Fig. 2B). Sensitivity analysis was performed via the leave-one-out method for achievement of target BP at 4–12 weeks comparing LDC with active comparators, and it was noted that heterogeneity persisted. On the other hand, Chow 2017 demonstrated an unusually large treatment effect and contributed disproportionately to heterogeneity for achievement of target BP at 4–12 weeks comparing LDC with placebo. However, sensitivity analysis excluding this trial yielded consistent results with substantially lower heterogeneity, supporting the robustness of the overall findings (OR: 4.86; 95% CI: 2.90–8.13; *P* < 0.00001; *I*^2^ = 17%) (Supplemental Digital Content Figure S1A, available at: https://links.lww.com/MS9/B412). Subgroup analysis according to regimen type demonstrated that both triple and quadruple LDC therapies significantly improved achievement of target BP compared with active comparators. Triple therapy showed an OR of 1.73 (95% CI: 1.21–2.48; *P* = 0.003; *I*^2^ = 77%), while quadruple therapy showed an OR of 2.78 (95% CI: 1.93–4.02; *P* < 0.00001; *I*^2^ = 0%). There was no statistically significant subgroup difference between triple and quadruple regimens (*P* = 0.07; *I*^2^ = 69.7%) (Supplemental Digital Content Figure S1B, available at: https://links.lww.com/MS9/B412). Similarly, subgroup analysis was performed on the basis of geographical location (high-income countries and low- and middle-income countries) and age of patients (<55 or >55 years). It was noted that the achievement of target BP at 4–12 weeks comparing LDC with active comparators remained statistically significant in all subgroups; however, heterogeneity did not decrease (Supplemental Digital Content Figure S1C and S1D, available at: https://links.lww.com/MS9/B412). At 6–12 months, three studies (1306 patients) demonstrated a sustained increase in the percentage of hypertensive patients achieving target BP compared with active comparators (OR: 1.76; 95% CI: 1.39–2.24; *P* < 0.00001; *I*^2^ = 0%, Fig. 2C).

**Figure 2.**
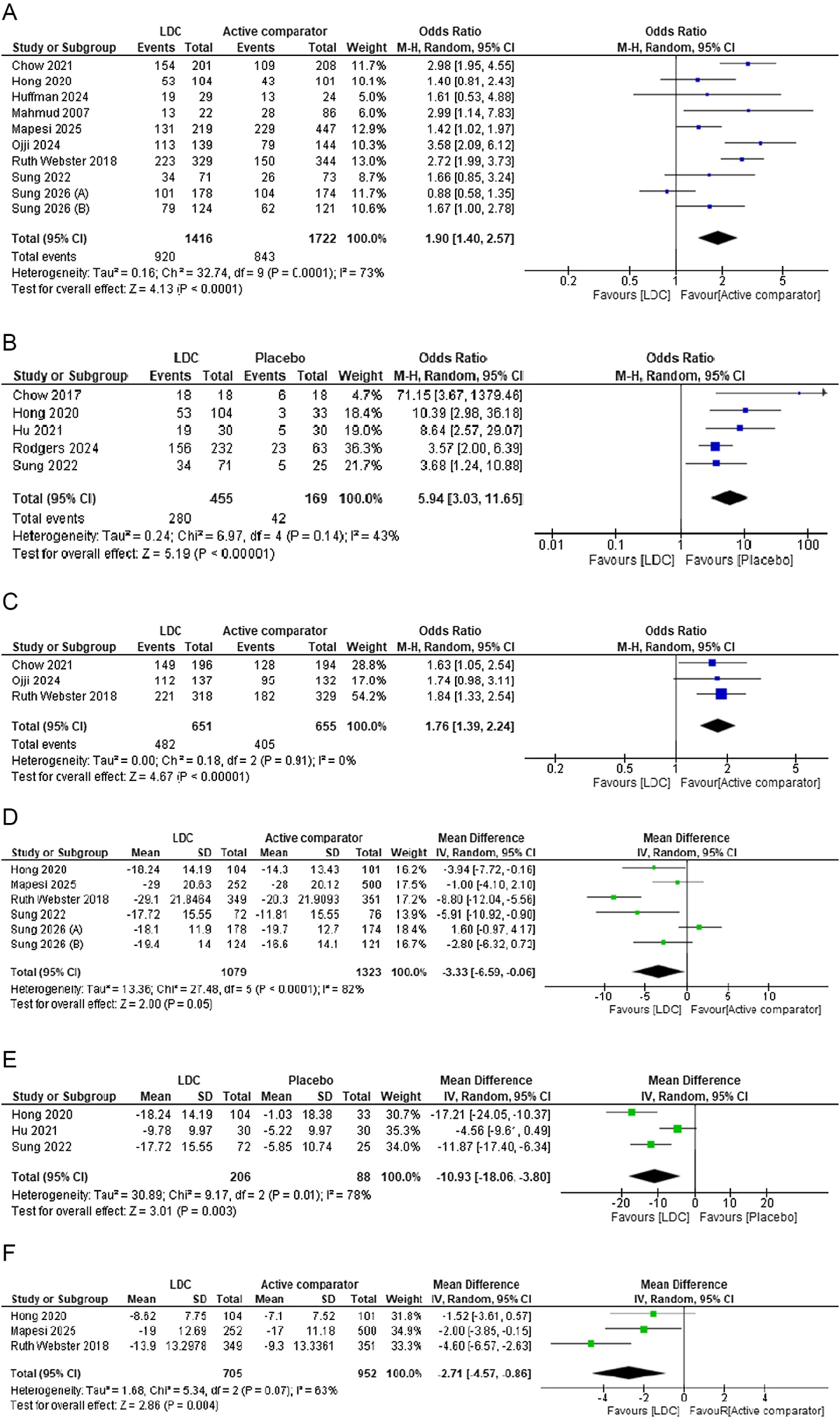
Forest plots demonstrating the primary outcomes.

#### Change in systolic and diastolic BP

According to our pooled analyses, LDC triple or quadruple therapy significantly reduced systolic BP compared with both active comparators and placebo in patients with hypertension. Across five studies (2402 patients) versus active comparators, the reduction was −3.33 mmHg (95% CI: −6.59 to −0.06; *P* = 0.05; *I*^2^ = 82%, Fig. 2D), and across three studies (294 patients) versus placebo, it was −10.93 mmHg (95% CI: −18.06 to −3.80; *P* = 0.003; *I*^2^ = 78%, Fig. 2E). Sensitivity analysis was performed via leave one out method for change in systolic BP comparing LDC and active comparator and it was noted that the heterogeneity remained persistent, however sensitivity analysis for change in systolic BP comparing LDC and placebo showed a reduction in heterogeneity from 78% to 29% after removing Hu 2021 (Supplemental Digital Content Figure S1E, available at: https://links.lww.com/MS9/B412). Subgroup analysis for change in systolic BP comparing LDC and active comparator on the basis of geographical location was performed and it was noted that the Low-middle income countries showed statistically significant reduction in BP, however the heterogeneity remained high in both subgroups (Supplemental Digital Content Figure S1F, available at: https://links.lww.com/MS9/B412). Pooled analysis of three studies (1657 patients) also demonstrated a significant reduction in diastolic BP with LDC therapy compared to active comparators (M.D. − 2.71 mmHg; 95% CI: −4.57 to −0.86; *P* = 0.004; *I*^2^ = 63%; Fig. 2F). Sensitivity analysis was performed via leave one out method for change in diastolic BP comparing LDC and active comparator and it was noted that the heterogeneity reduced from 63% to 0% after removing Ruth Webster 2018 (Supplemental Digital Content Figure S1G, available at: https://links.lww.com/MS9/B412).

### Secondary outcomes

#### LDC safety profile

Our findings indicate that LDC triple or quadruple therapy exhibited no significant difference in the incidence of adverse events compared with either active comparators across seven studies including 2355 patients (OR: 1.25; 95% CI: 0.76–2.05; *P* = 0.39; *I*^2^ = 77%; Fig. 3A) or with placebo across four studies including 466 patients (OR: 1.50; 95% CI: 0.75–2.98; *P* = 0.25; *I*^2^ = 29%; Fig. 3B). A sensitivity analysis for adverse events for LDC versus the active comparator was performed, and it was noted that the heterogeneity decreased from 77% to 30% after removing Zhao 2025 (Supplemental Digital Content Figure S1H, available at: https://links.lww.com/MS9/B412).

**Figure 3.**
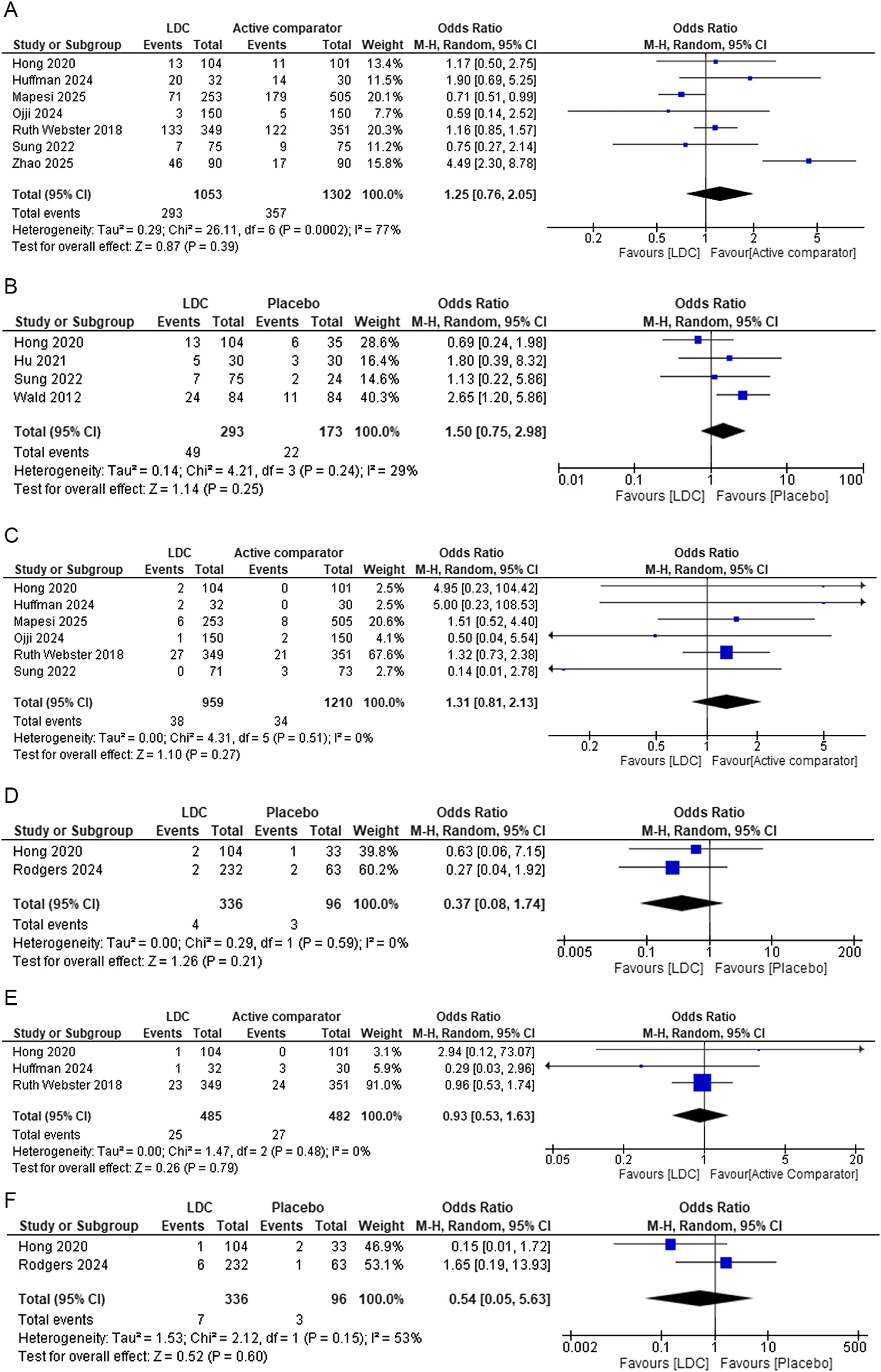
Forest plots demonstrating the secondary outcomes for the safety profile of LDC therapy.

Similarly, no significant difference was found in the incidence of ≥1 serious adverse event between LDC triple or quadruple therapy and either active comparators across six studies including 2169 patients (OR: 1.31; 95% CI: 0.81–2.13; *P* = 0.27; *I*^2^ = 0%; Fig. 3C) or placebo across two studies including 432 patients (OR: 0.37; 95% CI: 0.08–1.74; *P* = 0.21; *I*^2^ = 0%; Fig. 3D).

Lastly, our analysis demonstrated that LDC triple or quadruple therapy does not significantly affect study withdrawal due to adverse events compared with either active comparators or placebo. Across three studies (967 patients), there was no significant difference versus active comparators (OR: 0.93; 95% CI: 0.53–1.63; *P* = 0.79; *I*^2^ = 0%; Fig. 3E), and across two studies (432 patients) versus placebo (OR: 0.54; 95% CI: 0.05–5.63; *P* = 0.60; *I*^2^ = 53%; Fig. 3F). However, the absence of a statistically significant difference should be interpreted cautiously because the number of included trials and reported events was limited, and follow-up durations were generally short. Therefore, the available evidence provides limited power to detect uncommon or delayed adverse events.

#### Specific adverse events

Our results showed no significant difference in headache between LDC therapy and active comparators (seven studies, 2728 patients; OR: 0.98; 95% CI: 0.67–1.43; *P* = 0.93; *I*^2^ = 0%; Fig. 4A), whereas LDC therapy was associated with a significantly lower risk compared with placebo (two studies, 432 patients; OR, 0.27; 95% CI: 0.08–0.95; *P* = 0.04; *I*^2^ = 0%; Fig. 4B).

**Figure 4.**
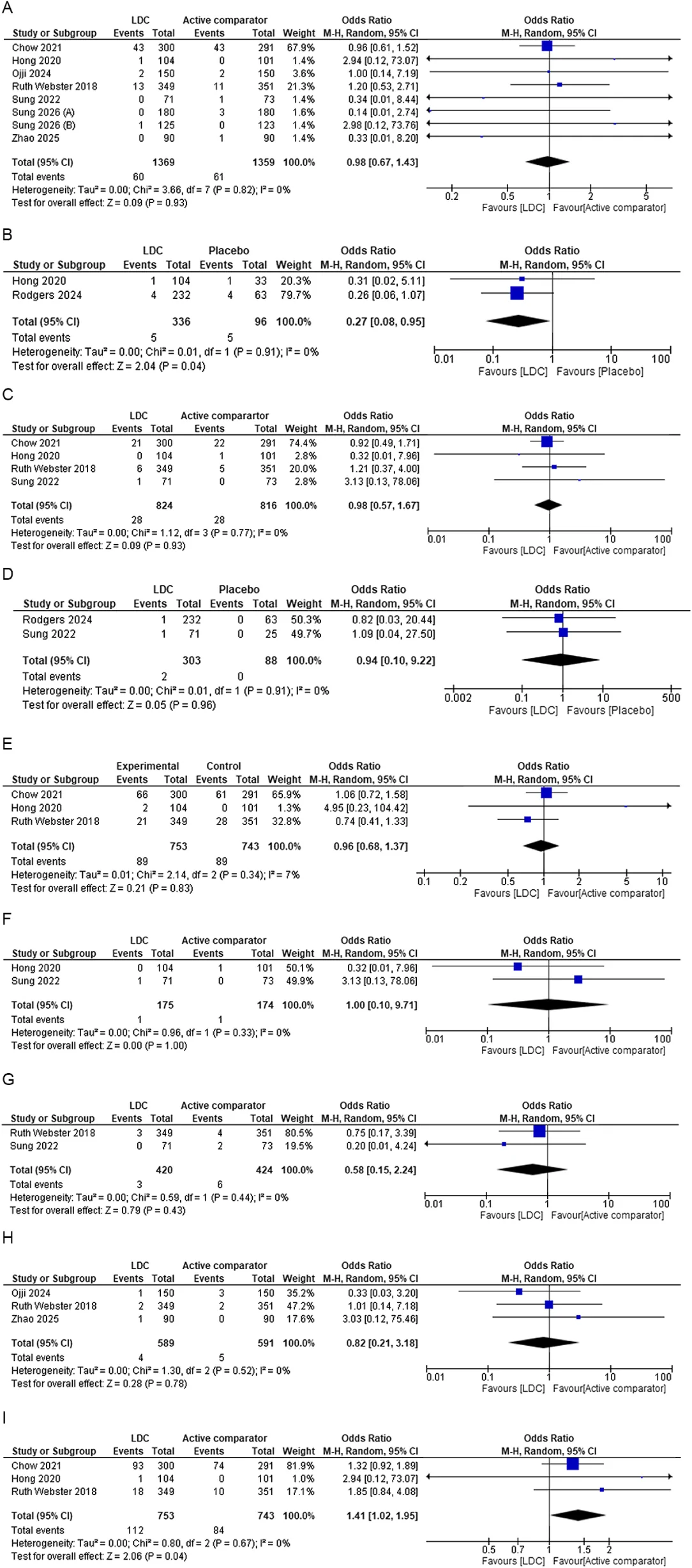
Forest plots demonstrating the secondary outcomes for specific adverse events with LDC therapy.

Our pooled analyses showed no significant difference in peripheral edema between LDC therapy and either active comparators (four studies, 1640 patients; OR: 0.98; 95% CI: 0.57–1.67; *P* = 0.93; *I*^2^ = 0%, Fig. 4C) or placebo (two studies, 391 patients; OR: 0.94; 95% CI: 0.10–9.22; *P* = 0.96; *I*^2^ = 0%, Fig. 4D).

There were no significant differences observed between LDC therapy and the active comparator for musculoskeletal pain (three studies, 1496 patients; OR: 0.96; 95% CI: 0.68–1.37; *P* = 0.83; *I*^2^ = 7%; Fig. 4E), pruritus (two studies, 349 patients; OR: 1.00; 95% CI: 0.10–9.71; *P* = 1.00; *I*^2^ = 0%; Fig. 4F), chest pain (two studies, 844 patients; OR: 0.58; 95% CI: 0.15–2.24; *P* = 0.43; *I*^2^ = 0%; Fig. 4G), and all types of hypotension (two studies, 1000 patients; OR 0.82; 95% CI: 0.21–3.18; *P* = 0.78; *I*^2^ = 0%; Fig. 4H). However, LDC was associated with a slightly higher risk of dizziness, syncope, or presyncope (three studies, 1496 patients; OR: 1.41; 95% CI: 1.02–1.95; *P* = 0.04; *I*^2^ = 0%; Fig. 4I).

#### Laboratory parameters

According to our findings, no significant differences were observed between LDC therapy and active comparators for serum levels of triglycerides (two studies, 905 patients; MD: 0.53 mg/dl; 95% CI: −12.11 to 13.16; *P* = 0.93; *I*^2^ = 0%; Fig. 5A), creatinine (three studies, 1205 patients; MD 0.01 mg/dl; 95% CI: −0.01 to 0.04; *P* = 0.17; *I*^2^ = 0%; Fig. 5B), AST (four studies, 1385 patients; MD 0.62 U/L; 95% CI: −0.74 to 1.99; *P* = 0.37; *I*^2^ = 0%; Fig. 5C), and ALT (four studies, 1385 patients; MD: 0.86 U/L; 95% CI: −1.15 to 2.87; *P* = 0.40; *I*^2^ = 15%; Fig. 5D).

**Figure 5.**
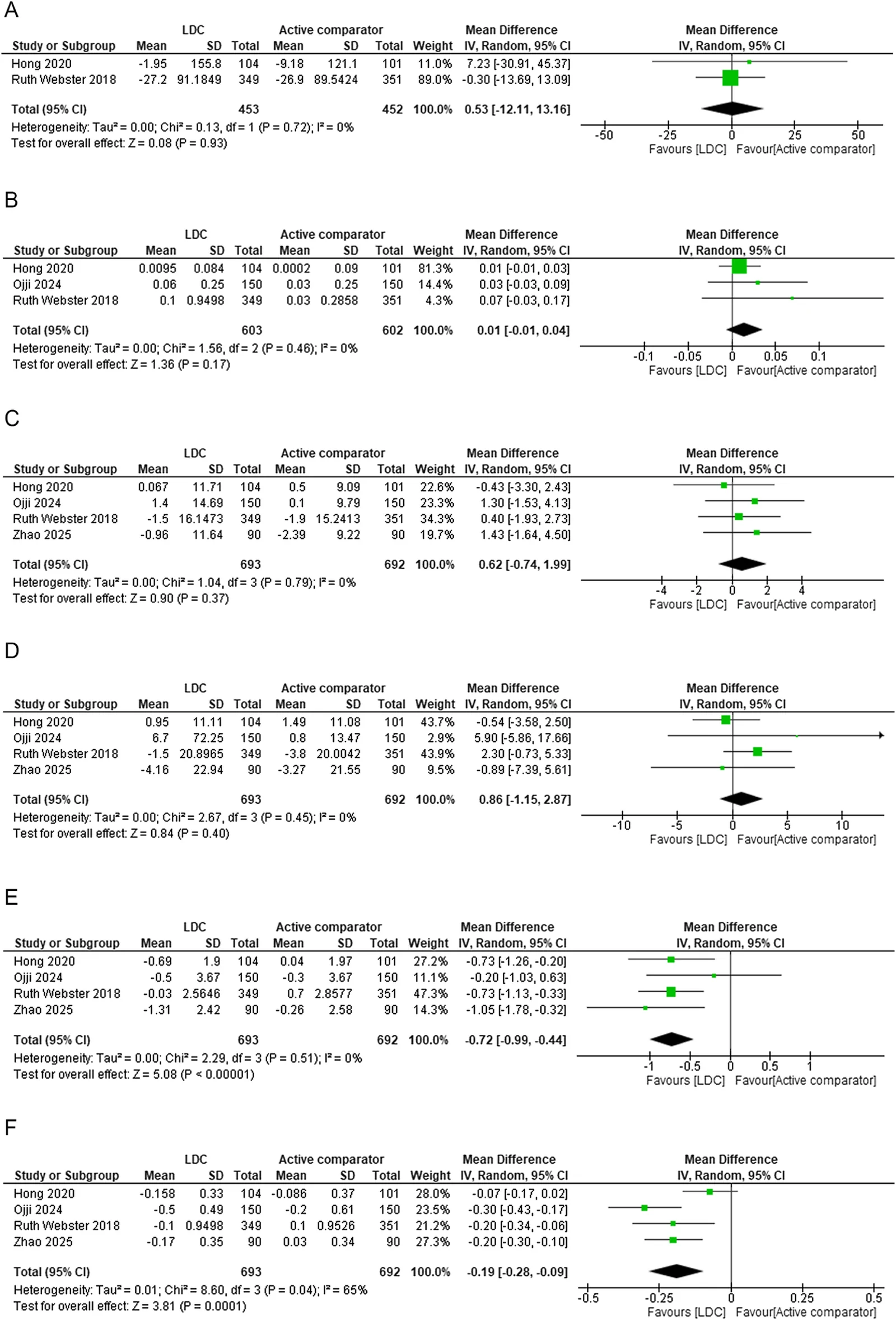
Forest plots demonstrating the secondary outcomes for changes in laboratory parameters with LDC therapy.

However, statistically significant but small reductions were observed in serum sodium levels (four studies, 1385 patients; MD: −0.72 mmol/L; 95% CI: −0.99 to −0.44; *P* < 0.00001; *I*^2^ = 0%; Fig. 5E) and potassium (four studies, 1385 patients; MD −0.19 mmol/L; 95% CI: −0.28 to −0.09; *P* = 0.0001; *I*^2^ = 65%; Fig. 5F), indicating minor electrolyte changes with LDC therapy compared with active comparators. Sensitivity analysis was performed via the leave-one-out method for the reduction in serum potassium levels when comparing LDC with active comparators, and it was noted that heterogeneity decreased from 65% to 0% after removing Hong 2020 (Supplemental Digital Content Figure S1I, available at: https://links.lww.com/MS9/B412).

#### Qualiy assessment

The ROB 2 tool was employed to assess the risk of bias. Six trials were judged to have low risk of bias, seven showed some concerns while one had high risk of bias. Overall, the RCTs were of moderate to good quality. Concerns were primarily related to deviations from intended interventions, missing outcome data, and selection of the reported result. The detailed results of quality assessment have been provided in Supplemental Digital Content Figure S2, available at: https://links.lww.com/MS9/B412.

#### Grading the evidence

The certainty of evidence was high for achievement of target BP (at 6–12 months) vs. active comparators. The primary efficacy outcomes of achievement of target BP (at 4–8 weeks) vs. active comparators and changes in systolic and diastolic BP vs. active comparators were rated as moderate due to high heterogeneity (*I*^2^ > 50%). Safety outcomes of overall adverse events and serious adverse events vs. active comparators were rated as moderate and low, respectively, due to sparse absolute event rates across the intervention and control groups (Supplemental Digital Content Table S3, available at: https://links.lww.com/MS9/B412).

## Discussion

This systematic review and meta-analysis of fourteen RCTs, including a total of 4082 patients with hypertension, demonstrated that initial LDC triple or quadruple therapy is significantly more effective than an active comparator (initial monotherapy or usual care) or placebo in achieving target BP control and producing greater reductions in both systolic and diastolic BP. These benefits were evident in the short term and were sustained over longer follow-up periods. These regimens were generally well tolerated, showing no meaningful increase in overall or serious adverse events. While minor electrolyte changes and a slightly higher risk of dizziness-related symptoms were observed with LDC therapy, the overall safety and tolerability profile remained favorable.

Our finding that a substantially higher proportion of patients achieve target BP with initial LDC triple or quadruple therapy at both short and longer follow-up periods aligns with a growing body of evidence favoring early multidrug regimens over traditional monotherapy or stepwise escalation. Subgroup analyses demonstrated consistent benefits for both triple and quadruple LDC regimens, with no statistically significant subgroup differences observed. Although quadruple therapy showed lower between-study heterogeneity, the overall treatment effects were directionally similar. Consistent with our finding, a recent meta-analysis of 1709 hypertensive patients showed that LDC therapy significantly increase the likelihood of reaching target BP compared with monotherapy or usual care across multiple trials, with benefits seen early and maintained over follow-up periods^[^36^]^. Given that a 5-mm Hg reduction in systolic BP is associated with approximately 10% reduction in major cardiovascular events, achieving guideline-recommended BP control is crucial^[^12,37,38^]^. Notably, secondary analyses of large trials such as Treatment for Immune Mediated Pathophysiology (TRIUMPH) and Quadruple Ultra-Low-Dose Treatment for Hypertension (QUARTET) have shown that patients initiated on LDC therapy not only reach target BP more frequently but also spend a greater proportion of time within target ranges, highlighting the durability and clinical relevance of early BP control^[^39,40^]^. This outcome is particularly important, as delayed or inadequate BP control is strongly associated with higher risks of stroke, myocardial infarction, and heart failure, whereas early achievement of target BP confers long-term cardiovascular protection^[^41,42^]^ By simultaneously targeting multiple pathophysiological mechanisms, initial LDC therapy may reduce therapeutic inertia, minimizes the need for repeated titration, and may improve adherence through simplified regimens, thereby reinforcing its role as an effective first-line strategy for optimizing hypertension control^[^12^]^.

In addition to improving the proportion of patients achieving target BP, our analysis demonstrated that initial LDC triple or quadruple therapy produced greater reductions in both systolic and diastolic BP compared with an active comparator or placebo, reinforcing evidence that multidrug regimens yield more potent hemodynamic effects than single-agent strategies. Similarly, complementary data from broader meta-analyses of antihypertensive combinations showed that dual and multidrug regimens significantly enhanced BP lowering compared with monotherapy, with additive effects that exceed those achieved by simply increasing the dose of a single agent^[^6,43^]^. The magnitude of pooled reductions in systolic and diastolic BP in our analysis was modest. While this finding should be interpreted cautiously and not solely on the basis of its statistical significance, these results also hold substantial clinical significance. Clinically, even modest reductions in systolic and diastolic BP are critically important, as both dimensions of BP independently predict cardiovascular morbidity and mortality, and even minimal reductions in BP have been linked to meaningful decreases in the risk of stroke and myocardial infarction^[^44^]^. Moreover, a population-based retrospective cohort of 13 350 patients demonstrated that patients receiving LDC therapy had lower rates of death or hospitalization for heart attack, heart failure, or stroke, likely related to improved adherence and sustained BP control^[^45^]^. Likewise, LDC triple-therapy trials conducted in South Asia and sub-Saharan Africa have consistently reported substantially higher BP control rates compared with standard stepped-care approaches, supporting the generalizability of this strategy across diverse healthcare settings and populations^[^21,27–29^]^. These findings underscore the importance of initiating multidrug strategies early in the treatment of hypertension to optimize BP control, improve adherence, reduce the risk of cardiovascular events, and ultimately mitigate the burden of uncontrolled hypertension on patients and healthcare systems^[^35^]^.

The safety profile of LDC triple or quadruple therapy observed in this meta-analysis is consistent with existing evidence and highlights its practical suitability as a first-line strategy for hypertension management. Similar to our findings, prior meta-analyses and large randomized trials have demonstrated that initiating LDC therapy does not significantly increase the risk of overall adverse events, serious adverse events, or treatment discontinuation compared with monotherapy or usual care, despite the use of multiple agents^[^9,16^]^. Trials evaluating fixed LDC therapy, including triple and quadruple regimens, have similarly reported favorable tolerability profiles, with adverse effects largely mild and rarely leading to treatment withdrawal^[^39,40^]^. Although a modest increase in dizziness-related symptoms has been noted in some studies, including ours, this has not translated into higher rates of serious adverse events or therapy discontinuation, highlighting its limited practical impact. From a clinical standpoint, the absence of a meaningful safety penalty reinforces the rationale for upfront LDC therapy, as improved BP control can be achieved without compromising tolerability, thereby allowing clinicians to implement guideline-recommended and multi-mechanistic treatment strategies with confidence for sustained long-term hypertension management. However, these findings should not be interpreted as definitive evidence of equivalent long-term safety across all patient populations. The relatively small number of trials, limited follow-up duration, and low event rates for several safety outcomes reduce the ability of the available evidence to detect uncommon or delayed adverse effects. Moreover, participants enrolled in randomized trials may differ from patients encountered in routine practice, particularly older adults, individuals with reduced renal reserve or CKD, and patients receiving multiple concomitant medications.

The laboratory findings indicate that LDC therapy is largely metabolically neutral and does not result in clinically meaningful biochemical disturbances. Renal function markers and hepatic enzymes remained comparable to active comparators across included trials, suggesting that the use of multiple agents at low doses does not confer additional renal or hepatic burden. Supporting this, both the TRIUMPH and QUARTET trials demonstrated stable renal and hepatic laboratory profiles with LDC triple and quadruple therapy, with only mild electrolyte changes that did not translate into clinically meaningful safety concerns^[^21,22^]^. Although modest reductions in serum sodium and potassium were observed, these changes were small, predictable, and unlikely to be clinically relevant, particularly in the absence of increased treatment discontinuation or serious adverse events. Previous systematic reviews and meta-analyses further confirm that such electrolyte shifts are minor and expected pharmacologically rather than indicative of cumulative toxicity^[^6,9^]^. From a patient-centered perspective, these findings are reassuring and support the safe implementation of initial LDC therapy with routine monitoring, reinforcing its suitability for achieving effective and sustained BP control in routine practice.

Despite the robust synthesis of available evidence, several limitations must be acknowledged. Many of the included trials had relatively short follow-up durations, which precluded the assessment of long-term cardiovascular outcomes. Considerable variability in drug combinations and dose ratios across studies also makes it difficult to identify optimal regimens. The majority of participants enrolled in these trials had uncomplicated hypertension, with limited representation of older adults, individuals with multiple comorbidities, or those at high cardiovascular risk; consequently, the generalizability of findings to these populations remains uncertain. Although sensitivity analyses were performed to address heterogeneity, residual variability arising from differences in follow-up periods, comparator therapies, study design, and geographic or demographic factors may still influence pooled estimates. Although most included trials were judged to have a low overall risk of bias, some studies raised concerns in specific domains, particularly regarding deviations from intended interventions, missing outcome data, and selective reporting. These limitations may introduce uncertainty into the pooled estimates and should be considered when interpreting the apparent benefits of LDC polypills. Furthermore, potential industry sponsorship or conflicts of interest, where present, may represent an additional source of concern that is not directly captured by the RoB 2 framework. Therefore, the findings should be interpreted as promising but not definitive. Additionally, the safety outcomes were underpowered, as they were derived from a small number of studies with limited event data, leading to wide CIs and uncertainty in effect estimates. Therefore, conclusions regarding safety should be interpreted with caution. Another important limitation is the reliance on surrogate outcomes, primarily BP, without sufficient data on hard clinical endpoints such as cardiovascular events, mortality, or long-term outcomes. Therefore, conclusions regarding long-term clinical benefit remain speculative and cannot be directly inferred from the available evidence. Finally, publication bias and selective reporting cannot be fully excluded, given that smaller studies with null or negative results may be underrepresented. Formal assessment of publication bias using funnel plots or Egger’s test was not performed, as none of the included outcomes had the minimum required number of studies (*n* ≥ 10), limiting the ability to reliably assess small-study effects. Moreover, only English-language studies were included; therefore, potential language bias cannot be excluded and may affect the comprehensiveness and generalizability of the results. Additionally, we did not search two major databases, Embase and Web of Science, due to limited access. A further limitation is that the available evidence provides limited insight into the real-world implementation of LDC polypill strategies, particularly in low-resource settings. Although these therapies may reduce pill burden and potentially improve adherence, their widespread adoption may be constrained by the cost and availability of fixed-dose combination products, differences in healthcare infrastructure, and inconsistent access to essential cardiovascular medications. Collectively, these limitations highlight the need for long-term, pragmatic, and diverse-population trials to more comprehensively define the efficacy, safety, and real-world applicability of initial LDC triple or quadruple therapy for hypertension. Future research should focus on large-scale randomized trials reporting clinically meaningful outcomes, including cardiovascular events and mortality. Additionally, individual participant data (IPD) meta-analyses may help better explore patient-level effects and long-term clinical implications.

## Conclusion

Our findings demonstrate that LDC therapy may increase the proportion of hypertensive patients achieving target BP and effectively lowers both systolic and diastolic BP compared with an active comparator or placebo. The therapy was well tolerated, with no statistically significant increase in adverse events, serious events, or treatment discontinuation, and laboratory parameters remained largely stable; however, these findings should be interpreted cautiously given the limited safety data. Although LDC polypill strategies are promising, the present evidence primarily demonstrates effects on blood-pressure surrogate outcomes and does not establish reductions in cardiovascular events or mortality. Accordingly, LDC polypills should be considered a potentially valuable treatment strategy rather than a universally established first-line standard for all patients with hypertension. Long-term cardiovascular outcomes, optimal regimen selection, and effectiveness in older adults or patients with multiple comorbidities remain understudied, highlighting the need for larger, longer-term, and more diverse trials to fully establish their role in routine clinical practice.

## Data Availability

All data analyzed in this systematic review and meta-analysis were derived from published randomized controlled trials and their supplementary materials. The extracted study-level dataset and analytic files (including the search strategy, screening decisions, and meta-analysis calculations) are available from the corresponding author upon reasonable request. No new individual participant data were generated or analyzed in this study.

## Research registration unique identification number (UIN)

This systematic review and meta-analysis were registered in PROSPERO (Registration No. CRD420261323534).

## Reporting guidelines

This study was conducted and reported in accordance with the PRISMA 2020 guidelines.
